# Supplementary material for: From genes to patterns: a framework for modeling the emergence of embryonic development from transcriptional regulation
Source: Front Cell Dev Biol. 2025 Mar 20;13:1522725. doi: 10.3389/fcell.2025.1522725 (PMC11966961; doi:10.3389/fcell.2025.1522725)
Supplement: Supplementary file 1 [file Supplementaryfile5.docx]

**Text S2**

**Computer Simulation 1.** Modeling the Kinetics of a Simple Chemical Reaction Between A and B to Form C.

The results of this computer simulation are shown in Figure S1-A'.

% This MATLAB script models the kinetics of a chemical reaction where chemicals A and B react to produce chemical C.

% The reaction kinetics are described using ordinary differential equations (ODEs) that account for the consumption

% of chemicals A and B at a rate proportional to their concentrations, and the formation of chemical C at the same rate.

% Initial concentrations for each chemical are defined, and the ODE solver ode45 is used to simulate the reaction

% dynamics over a specified time range. The output is a plot of the concentrations of A, B, and C over time, illustrating

% the progression of the reaction and the interplay between reactant consumption and product formation.

% Clear workspace and command window to ensure a clean start

clear

clc

% Define the time range for the simulation from 0 to 10 with increments of 0.01

t = 0:.01:10;

% Initial concentrations of chemicals A, B, and C

A0 = 2;

B0 = 1;

C0 = 5;

% Reaction rate constant

k = 1;

% Solve the system of differential equations using ode45

% odefun - function defining the system of equations

% t - time range for the solution

% [A0 B0 C0 k] - initial conditions for A, B, C, and k

[T, y] = ode45(@odefun, t, [A0 B0 C0 k]);

% Extracting the solution for each chemical's concentration over time

A = y(:,1);

B = y(:,2);

C = y(:,3);

% Plotting the concentrations of A, B, and C over time

plot(T, [A B C])

xlabel 'time' % Label for the x-axis

ylabel 'concentration' % Label for the y-axis

legend('A', 'B', 'C') % Legend for each line representing a chemical

%%%%% Differential Equation %%%%%%

% Definition of the system of differential equations to model the chemical reactions

function dy = odefun(t, y)

% Extracting individual concentrations from the vector y

A = y(1);

B = y(2);

C = y(3);

k = y(4); % Reaction rate constant

% Defining the differential equations representing the reaction rates

dA = -k*A*B; % Rate of change of A, representing consumption of A

dB = -k*A*B; % Rate of change of B, representing consumption of B

dC = k*A*B; % Rate of change of C, representing formation of C

% Aggregating the differential equations into a vector to return

dy = [dA dB dC 0]';

end

**Computer Simulation 2.** Modeling Reversible Chemical Reaction Dynamics Towards Equilibrium.

The results of this computer simulation are shown in Figure S1-B'.

% This MATLAB script models the reversible kinetics of a chemical reaction where

% chemicals A and B react to form chemical C with a forward rate constant k, and

% chemical C decomposes back into chemicals A and B with a reverse rate constant k'.

% The reaction kinetics are described using ordinary differential equations (ODEs)

% that account for the formation and consumption of chemicals based on their

% concentrations. Initial concentrations for each chemical are defined, and the ODE

% solver ode45 is used to simulate the reaction dynamics over a specified time range

% until t=10. The output is a plot of the concentrations of A, B, and C over time,

% illustrating the progression of the reaction towards equilibrium within this timeframe.

% Clear workspace and command window to ensure a clean start

clear

clc

% Define the time range for the simulation to t=10

t = 0:.01:10; % Updated time range

% Initial concentrations of chemicals A, B, and C

A0 = 2;

B0 = 1;

C0 = 0;

% Reaction rate constants

k = 1; % Forward reaction rate constant

k_prime = 0.5; % Reverse reaction rate constant

% Solve the system of differential equations using ode45

% The lambda function passes additional parameters k and k_prime to odefun

[T, y] = ode45(@(t, y) odefun(t, y, k, k_prime), t, [A0 B0 C0]);

% Extracting the solution for each chemical's concentration over time

A = y(:,1);

B = y(:,2);

C = y(:,3);

% Plotting the concentrations of A, B, and C over time

plot(T, [A B C])

xlabel 'time' % Label for the x-axis

ylabel 'concentration' % Label for the y-axis

title('Concentrations of A, B, and C Over Time Towards Equilibrium (t=10)')

legend('A', 'B', 'C') % Legend for each line representing a chemical

% Definition of the system of differential equations to model the reversible chemical reactions

function dy = odefun(t, y, k, k_prime)

% Extracting individual concentrations from the vector y

A = y(1);

B = y(2);

C = y(3);

% Defining the differential equations for the forward and reverse reactions

dA = -k*A*B + k_prime*C; % Rate of change of A due to both forward and reverse reactions

dB = -k*A*B + k_prime*C; % Rate of change of B due to both forward and reverse reactions

dC = k*A*B - k_prime*C; % Rate of change of C due to both forward and reverse reactions

% Aggregating the differential equations into a vector to return

dy = [dA; dB; dC];

end

**Computer Simulation 3.** Modeling Gene Activation by a Single Activator with Varying Transcription Factor Binding Dynamics.

The results of this computer simulation are shown in Fig. S2-A (fast TF binding dynamics; high k1 and k_1), and Fig. S2-B (slow TF binding dynamics; low k1 and k_1). Recommended values for binding constants for slow dynamics are k1=k_1=1. Recommended values for binding constants for fast dynamics are k1=k_1=.01.

% This script models gene regulation, simulating the dynamic processes of gene expression

% over time. It employs a differential equation model to describe the interaction between

% a Transcription Facotr (X), an active DNA state (D1), an output transcript (Y), and the total DNA

% concentration (DT), under the influence of various reaction rate constants (k1, k-1, kt, lambda).

%

% Symbols:

% X - Concentration of TF X that influences DNA state.

% D1 - Concentration of the DNA in an active state, capable of transcription.

% Y - Concentration of the output mRNA produced from the active DNA state.

% DT - Total concentration of DNA, assumed to be constant.

% k1 - Rate constant for the activation of DNA by the regulatory protein X.

% k-1 - Rate constant for the deactivation of the active DNA state back to its inactive state.

% kt - Transcription rate constant, representing the rate at which Y is produced from the active DNA state.

% lambda - Degradation rate constant for the output protein Y.

% Clear workspace and command window to ensure a clean start

clear

clc

% Define the time range for the simulation

t = 0:.01:10;

% Initial conditions

X0 = 10; % Initial concentration of X

D1_0 = 0; % Initial concentration of D1

Y0 = 0; % Initial concentration of Y

DT = 1; % Total DNA concentration

% Reaction rate constants

k1 = 1; % Activation rate constant (1 for fast, and .01 for slow)

k_1 = 1; % Deactivation rate constant (1 for fast, and .01 for slow)

kt = 5; % Transcription rate constant

lambda = 1; % Degradation rate constant

% Initial conditions array for ODE solver

initial_conditions = [X0 D1_0 Y0];

% Solve the system of differential equations using ode45

[T, y] = ode45(@(t, y) odefun(t, y, DT, k1, k_1, kt, lambda), t, initial_conditions);

% Plotting the results

plot(T, [y(:,1) y(:,3) y(:,2)])

xlabel('time')

ylabel('concentration')

legend('X', 'Y', 'D1')

grid on

% Function defining the system of differential equations for gene regulation

function dy = odefun(t, y, DT, k1, k_1, kt, lambda)

X = y(1); % X concentration

D1 = y(2); % D1 concentration

Y = y(3); % Y concentration

% Differential equations

dD1 = k1 * (DT - D1) * X - k_1 * D1; % Change in D1

dX = -dD1; % Change in X, assuming conservation

dY = kt * D1 - lambda * Y; % Production of Y and degradation

% Compile derivatives into a vector

dy = [dX; dD1; dY];

end

**Computer Simulation 4**. Modeling Gene Activation Using Michaelis-Menten Kinetics.

The results of this computer simulation are shown in Figure S2-C.

% This script models gene regulation using the Michaelis-Menten kinetics to describe the

% interaction between a Transcription Factor (TF) X and the regulatory region of gene Y.

% X represents the concentration of the TF that can bind to the regulatory region,

% initiating the transcription of gene Y. Y represents the mRNA concentration of gene Y,

% indicating the level of gene expression. The model incorporates Michaelis-Menten kinetics

% for the transcription process and includes a degradation term for mRNA to simulate its decay.

% Clear workspace and command window to ensure a clean start

clear

clc

% Define the time range for the simulation

t = 0:.01:10;

% Initial conditions

X0 = 10; % Initial concentration of TF X

Y0 = 0; % Initial mRNA concentration of gene Y

% Reaction rate constants

k1 = 10; % Forward reaction rate constant for TF binding

k_1 = 10; % Reverse reaction rate constant for TF unbinding

% Michaelis-Menten constant

Km = k_1 / k1; % Km, representing the affinity of TF X to the regulatory region of gene Y

% Production and degradation parameters

alpha = 5; % Rate constant for mRNA production of gene Y

lambda = 1; % Degradation rate constant for mRNA of gene Y

% Array of initial conditions for the ODE solver

initial_conditions = [X0 Y0];

% Solve the system of differential equations using ode45

% Note: Additional parameters (Km, alpha, lambda) are passed directly into odefun

[T, y] = ode45(@(t, y) odefun(t, y, Km, alpha, lambda), t, initial_conditions);

% Plotting TF X concentration and Y mRNA concentration over time

plot(T, [y(:,1) y(:,2)])

xlabel('time')

ylabel('concentration')

ylim([0 11])

legend('X','Y')

grid on

% Function defining the differential equation based on Michaelis-Menten kinetics

function dy = odefun(t, y, Km, alpha, lambda)

% Variables in the system

X = y(1); % Concentration of TF X (constant in this model)

Y = y(2); % mRNA concentration of gene Y

% Differential equation for the change in mRNA concentration of Y

% Incorporates production (alpha * X / (Km + X)) and degradation (-lambda * Y)

dY = alpha * X / (Km + X) - lambda * Y;

% Since X is constant, its derivative is zero. Km, alpha, and lambda are parameters, not variables.

dy = [0; dY];

end

**Computer Simulation 5.** Input-Output Relationship Between Activator Concentration and Gene Expression Using Michaelis-Menten Dynamics.

The results of this computer simulation are shown in Figure 1B.

% This script models the input-output relationship between the concentration

% of a transcription factor (TF) X acting as an activator of gene Y, and the

% steady state concentration of Y mRNA according to Michaelis-Menten dynamics.

% It plots the relationship between varying concentrations of X and the resulting

% steady state concentration of Y mRNA. This model assumes that the activation

% of gene Y by TF X follows Michaelis-Menten kinetics.

clear % Clears all the variables from the workspace to ensure a clean environment

clc % Clears the command window for clarity

% Define the range of X concentrations (TF X acting as an activator of gene Y)

X = 0:0.1:10; % TF concentration range from 0 to 10 with increments of 0.1

% Michaelis-Menten constant, representing the TF concentration at which the reaction

% rate is half of its maximum value. It is a measure of the affinity of the enzyme

% (or in this case, the TF) for its substrate (the gene Y).

Km = 5;

% Maximum rate achieved by the system, at saturating substrate concentration

alpha = 1;

% Degradation rate of Y mRNA

lambda = 1;

% Calculate the steady state concentration of Y mRNA using the Michaelis-Menten equation.

% The equation models the rate of enzymatic reactions, here used to describe the activation

% of gene Y by TF X. It assumes that the rate of mRNA production is directly proportional

% to the concentration of the TF, modulated by the degradation rate (lambda).

Y = alpha / lambda * (X / Km) ./ (1 + (X / Km));

% Plotting the relationship between the concentration of TF X and the steady state

% concentration of Y mRNA. This plot helps visualize how changes in the TF concentration

% influence the gene's expression level.

plot(X, Y)

xlabel('Concentration of TF X') % Label for the x-axis

ylabel('Steady state concentration of Y mRNA') % Label for the y-axis

title('TF X Activation of Gene Y according to Michaelis-Menten Dynamics') % Title of the plot

**Computer Simulation 6.** Input-Output Relationship Between Repressor Concentration and Gene Expression.

The results of this computer simulation are shown in Figure 1D.

% This script models the input-output relationship between the concentration

% of a transcription factor (TF) X acting as a repressor of gene Y, and the

% steady state concentration of Y mRNA. The model incorporates Michaelis-Menten-like dynamics

% to describe the repression, with the additional consideration of mRNA degradation rate (lambda).

% It calculates and plots the steady state concentration of Y mRNA as a function of

% the concentration of the repressor X.

clear % Clears all the variables from the workspace to ensure a clean environment.

clc % Clears the command window for clarity.

% Define the range of X concentrations (TF X acting as a repressor of gene Y).

X = 0:0.1:10; % TF concentration range from 0 to 10 with increments of 0.1.

% Michaelis-Menten constant, representing the TF concentration at which the

% repressive effect is half of its maximum value.

Km = 5;

% Maximum possible expression level of Y in the absence of the repressor X.

alpha = 1;

% Degradation rate of Y mRNA, affecting the steady state concentration.

lambda = 1;

% Calculate the steady state concentration of Y mRNA.

Y = (alpha / lambda) * (1 ./ (1 + X/Km));

% Plotting the relationship between the concentration of TF X and the steady state

% concentration of Y mRNA to visualize the repressive effect of X on Y's expression.

plot(X, Y)

xlabel('Concentration of TF X') % Label for the x-axis.

ylabel('Steady state concentration of Y mRNA') % Label for the y-axis.

title('TF X Repression of Gene Y: Impact of Repression and mRNA Degradation') % Title of the plot.

**Computer Simulation 7.** Effect of Cooperative Binding on Gene Activation Using the Hill Function.

The results of this computer simulation are shown in Fig. 1F (*n*=2) and Fig. 1G (*n*=5).

% This script models the input-output relationship between the concentration

% of a transcription factor (TF) X and the steady-state concentration of a gene's

% mRNA, where TF X binds to the gene's regulatory element with cooperativity.

% The relationship is modeled using the Hill function, which captures the effect

% of cooperativity in TF binding. The script calculates the steady-state

% concentration of the mRNA as a function of varying concentrations of TF X and

% plots this relationship.

clear % Clears all the variables from the workspace to ensure a clean environment.

clc % Clears the command window for clarity.

% Define the range of X concentrations (TF X concentration).

X = 0:0.1:10; % TF concentration range from 0 to 10 with increments of 0.1.

% Dissociation constant, Kx, representing the TF concentration at which the binding

% to the regulatory element is half its maximum. It is a measure of the affinity of

% TF X for its binding site.

Kx = 5;

% Maximum rate of gene expression (or maximum mRNA concentration) when the regulatory

% element is fully occupied by TF X.

alpha = 1;

% Degradation rate of mRNA, affecting how quickly the mRNA concentration decreases

% over time.

lambda = 1;

% Cooperativity coefficient, n, indicating the degree of cooperativity in TF X binding.

% A higher n value indicates a greater cooperative effect, where small changes in TF X

% concentration can lead to significant changes in gene expression.

n = 5;

% Calculation of the steady-state concentration of mRNA using the Hill equation. The Hill

% equation models the effect of TF concentration on gene expression, taking into account

% the cooperativity of binding. The expression level increases with TF concentration in a

% sigmoidal manner, reflecting cooperative binding dynamics.

Y = alpha / lambda * (X / Kx).^n ./ (1 + (X / Kx).^n);

% Plotting the relationship between the concentration of TF X and the steady state

% concentration of mRNA. This plot helps visualize the cooperative binding effect of TF X

% on the gene's expression, as described by the Hill function.

plot(X, Y)

xlabel('Concentration of TF X') % Label for the x-axis.

ylabel('Steady state concentration of mRNA') % Label for the y-axis.

title('Gene Expression Modulation by TF X: A Hill Function Representation') % Title of the plot.

**Computer Simulation 8.** Modeling Gene Activation by Two ORed Activators Using Hill Functions.

The results of this computer simulation are shown in Fig. 2B.

% This script models and plots the input-output relationship for a gene Y that has two

% transcription factors (TFs), A and B, regulating it with an OR logic. The model uses Hill

% functions to describe the regulatory effects of A and B on Y. The input is the concentrations

% of A and B, and the output is the steady-state concentration of Y. The plot is a 3D surface

% showing how variations in concentrations of A and B affect the activation of Y.

% Create a mesh grid of A and B concentrations for plotting.

[A, B] = meshgrid(0:.1:2, 0:.1:2); % Concentration ranges from 0 to 2 with 0.1 increments.

% Parameters for the model: dissociation constants and cooperativity coefficients.

ka = 1; % Dissociation constant for A.

kb = 1; % Dissociation constant for B.

na = 10; % Cooperativity coefficient for A.

nb = 10; % Cooperativity coefficient for B.

% Calculation of the steady-state concentration of Y, given the concentrations of A and B,

% using the Hill function to model the OR logic regulatory effect.

Y_steady_state = (A/ka).^na./(1+(A/ka).^na) + ((B/kb).^nb)./(1+(B/kb).^nb);

% Plotting the 3D surface to visualize the relationship between A, B concentrations,

% and the steady-state concentration of Y. This visual representation helps illustrate

% the effect of TF concentrations on gene Y's activation under OR logic.

surf(A, B, Y_steady_state, 'EdgeColor', 'none') % Plot without edge color for smoother appearance.

xlabel('A') % Label for the x-axis, representing concentration of A.

ylabel('B') % Label for the y-axis, representing concentration of B.

zlabel('Y steady state') % Label for the z-axis, representing steady-state concentration of Y.

colorbar

print(gcf, '-depsc', 'Hill_OR.eps', '-painters') % Save in editable format

**Computer Simulation 9.** Modeling Gene Activation by Two Competitive ORed Activators.

The results of this computer simulation are shown in Fig. 2D.

% This script models and plots the input-output relationship for a gene Y that has two

% transcription factors (TFs), A and B, regulating it with a competitive OR logic. The model

% uses a modified Hill function to describe the regulatory effects of A and B on Y, allowing

% for competition between A and B in activating Y. The input is the concentrations of A and B,

% and the output is the steady-state concentration of Y. The plot is a 3D surface showing how

% the competition between A and B influences the activation of Y, with a color scale indicating

% the concentration of Y.

% Create a mesh grid of A and B concentrations for plotting.

[A, B] = meshgrid(0:.1:2, 0:.1:2); % Concentration ranges from 0 to 2 with 0.1 increments.

% Parameters for the model: dissociation constants and cooperativity coefficients.

ka = 1; % Dissociation constant for A.

kb = 1; % Dissociation constant for B.

na = 10; % Cooperativity coefficient for A.

nb = 10; % Cooperativity coefficient for B.

% Calculation of the steady-state concentration of Y, given the concentrations of A and B,

% using a modified Hill function to model the competitive OR logic regulatory effect.

% A and B compete in activating Y, affecting each other's influence.

Y_steady_state = ((A/ka).^na + (B/kb).^nb) ./ (1 + (A/ka).^na + (B/kb).^nb);

% Plotting the 3D surface to visualize the relationship between A, B concentrations,

% and the steady-state concentration of Y. This visual representation helps illustrate

% the competitive OR logic effect of TF concentrations on gene Y's activation.

surfHandle = surf(A, B, Y_steady_state, 'EdgeColor', 'none'); % Plot without edge color for smoother appearance.

xlabel('A') % Label for the x-axis, representing concentration of A.

ylabel('B') % Label for the y-axis, representing concentration of B.

zlabel('Y steady state') % Label for the z-axis, representing steady-state concentration of Y.

% Add a color scale to the plot to indicate the steady-state concentration of Y.

colorbar;

ylabel(colorbar, 'Y steady-state concentration') % Label the color scale to indicate what it represents.

print(gcf, '-depsc', 'Hill_CompetitiveOR.eps', '-painters') % Save in editable format

**Computer Simulation 10.** Modeling Gene Activation by Two ANDed Activators.

The results of this computer simulation are shown in Fig. 2F.

% This script models and plots the input-output relationship for a gene Y that has two

% transcription factors (TFs), A and B, regulating it with an AND logic. The model uses Hill

% functions to describe the regulatory effects of A and B on Y, requiring both A and B to

% be present for activation. The input is the concentrations of A and B, and the output is

% the steady-state concentration of Y. The plot is a 3D surface showing how the presence of

% both A and B is necessary to activate Y, with a color scale indicating the concentration of Y.

% Create a mesh grid of A and B concentrations for plotting.

[A, B] = meshgrid(0:.1:2, 0:.1:2); % Concentration ranges from 0 to 2 with 0.1 increments.

% Parameters for the model: dissociation constants and cooperativity coefficients.

ka = 1; % Dissociation constant for A.

kb = 1; % Dissociation constant for B.

na = 10; % Cooperativity coefficient for A.

nb = 10; % Cooperativity coefficient for B.

% Calculation of the steady-state concentration of Y, given the concentrations of A and B,

% using the Hill function to model the AND logic regulatory effect. Both A and B must be

% present for Y to be activated significantly.

Y_steady_state = ((A/ka).^na ./ (1 + (A/ka).^na)) .* ((B/kb).^nb ./ (1 + (B/kb).^nb));

% Plotting the 3D surface to visualize the relationship between A, B concentrations,

% and the steady-state concentration of Y. This visual representation illustrates

% the effect of TF concentrations on gene Y's activation under AND logic.

surfHandle = surfc(A, B, Y_steady_state, 'EdgeColor', 'none'); % Plot without edge color for smoother appearance.

xlabel('A') % Label for the x-axis, representing concentration of A.

ylabel('B') % Label for the y-axis, representing concentration of B.

zlabel('Y steady state') % Label for the z-axis, representing steady-state concentration of Y.

% Add a color scale to the plot to indicate the steady-state concentration of Y.

colorbar;

ylabel(colorbar, 'Y steady-state concentration') % Label the color scale to indicate what it represents.

print(gcf, '-depsc', 'Hill_AND.eps', '-painters') % Save in editable format

**Computer Simulation 11.** Modeling a Positive Feedback Loop Between Two Activator Genes.

The results of this computer simulation are shown in Fig. 3B’ (X0=0.5, Y0=0.8), and 3B’’ (X0=1.2, Y0=1.7).

% This script models a two-gene regulatory network (GRN) where each gene is

% activating the other, forming a positive feedback loop. The dynamics of the

% system are explored over time, showing how the concentrations of gene products

% X and Y change. The model is solved numerically using ordinary differential

% equations (ODEs) to simulate the activation dynamics under positive feedback.

clear % Clears all variables from the workspace for a clean start.

clc % Clears the command window for clarity.

% Define the time range for the simulation.

t = 0:0.01:10; % Time range from 0 to 10 with 0.01 time step increments.

% Initial concentrations of gene products X and Y.

X0 = 1.2; % Initial concentration of gene product X.

Y0 = 1.7; % Initial concentration of gene product Y.

% Parameters for the model: dissociation constants, cooperativity coefficients,

% maximum rate of gene expression, and degradation rate.

Kxy = 1; % Dissociation constant for the effect of Y on X.

Kyx = 1; % Dissociation constant for the effect of X on Y.

nxy = 5; % Cooperativity coefficient for the effect of Y on X.

nyx = 5; % Cooperativity coefficient for the effect of X on Y.

alpha = 2; % Maximum rate of gene expression when fully activated.

lambda = 1; % Degradation rate of gene products.

% Group initial conditions and parameters into a vector for ode45.

initial_conditions = [X0, Y0, Kxy, Kyx, nxy, nyx, alpha, lambda];

% Solve the system of differential equations using ode45.

[T, y] = ode45(@odefun, t, initial_conditions);

% Plotting the results: concentrations of X and Y over time.

plot(T, [y(:, 1:2)])

xlabel('time') % Label for the x-axis.

ylabel('concentration') % Label for the y-axis.

legend('X', 'Y') % Legend to distinguish the curves for X and Y.

ylim([0 2]) % Set the y-axis limits for better visualization.

% grid on % Ensure the grid is on for the plot.

% Definition of the system of differential equations to be solved.

function dy = odefun(t, y)

% Unpack the variables from the vector y.

X = y(1);

Y = y(2);

Kxy = y(3);

Kyx = y(4);

nxy = y(5);

nyx = y(6);

alpha = y(7);

lambda = y(8);

% Differential equations describing the changes in concentrations of X and Y.

% The equations model the positive feedback loop where X activates Y and Y activates X.

dX = alpha * (Y/Kxy)^nxy / (1 + (Y/Kxy)^nxy) - lambda * X;

dY = alpha * (X/Kyx)^nyx / (1 + (X/Kyx)^nyx) - lambda * Y;

% Return the derivatives of X and Y.

dy = [dX; dY; 0; 0; 0; 0; 0; 0];

end

**Computer Simulation 12.** Modeling a Mutual Repression Loop Between Two Repressor Genes.

The results of this computer simulation are shown in Fig. 3C’ (X0=0.8, Y0=1.5), and 3C’’ (X0=1.5, Y0=0.8).

% This script models a two-gene regulatory network (GRN) where each gene product

% represses the other, forming a positive feedback loop through repression. The

% dynamics of the system are explored over time to show how the concentrations of

% gene products X and Y change due to the mutual repression. The model is solved

% numerically using ordinary differential equations (ODEs) to simulate the

% repression dynamics under positive feedback.

clear % Clears all variables from the workspace for a clean start.

clc % Clears the command window for clarity.

% Define the time range for the simulation.

t = 0:0.01:10; % Time range from 0 to 10 with 0.01 time step increments.

% Initial concentrations of gene products X and Y.

X0 = 0.8; % Initial concentration of gene product X.

Y0 = 1.5; % Initial concentration of gene product Y.

% Parameters for the model: dissociation constants, cooperativity coefficients,

% maximum rate of gene expression, and degradation rate.

Kxy = 1; % Dissociation constant for the effect of Y on X repression.

Kyx = 1; % Dissociation constant for the effect of X on Y repression.

nxy = 5; % Cooperativity coefficient for the effect of Y on X repression.

nyx = 5; % Cooperativity coefficient for the effect of X on Y repression.

alpha = 2; % Maximum rate of gene expression when not repressed.

lambda = 1; % Degradation rate of gene products.

% Group initial conditions and parameters into a vector for ode45.

initial_conditions = [X0, Y0, Kxy, Kyx, nxy, nyx, alpha, lambda];

% Solve the system of differential equations using ode45.

[T, y] = ode45(@odefun, t, initial_conditions);

% Plotting the results: concentrations of X and Y over time.

plot(T, [y(:, 1:2)])

xlabel('time') % Label for the x-axis.

ylabel('concentration') % Label for the y-axis.

legend('X', 'Y') % Legend to distinguish the curves for X and Y.

ylim([0 2.5]) % Set the y-axis limits for better visualization.

% grid on % Ensure the grid is on for the plot.

% Definition of the system of differential equations to be solved.

function dy = odefun(t, y)

% Unpack the variables from the vector y.

X = y(1);

Y = y(2);

Kxy = y(3);

Kyx = y(4);

nxy = y(5);

nyx = y(6);

alpha = y(7);

lambda = y(8);

% Differential equations describing the changes in concentrations of X and Y due to mutual repression.

% The equations model the positive feedback loop where the repression of one gene by the other indirectly

% promotes its own production.

dX = alpha / (1 + (Y/Kxy)^nxy) - lambda * X;

dY = alpha / (1 + (X/Kyx)^nyx) - lambda * Y;

% Return the derivatives of X and Y, indicating the change in their concentrations over time.

dy = [dX; dY; 0; 0; 0; 0; 0; 0];

end

**Computer Simulation 13**. Modeling a Negative Feedback Loop Between an Activator and a Repressor Gene.

The results of this computer simulation are shown in Fig. 3D’.

% ********************************************************

% This program models a two-gene negative feedback loop on transcription.

% In this type of circuit, two genes repress each other's

% transcription, leading to oscillatory or stable mRNA levels.

%

% Key Variables:

% * X, Y: Concentrations of mRNA transcripts

% * Kxy, Kyx: Dissociation constants (influence repression strength)

% * nxy, nyx: Hill coefficients (determine cooperativity in repression)

% * alpha: Maximum transcription rate

% * lambda: Degradation rate of mRNA transcripts

% ********************************************************

clear;

clc;

grid on;

% Parameter Definitions

t = 0:0.01:10; % Time span

X0 = 2; % Initial concentration of mRNA X

Y0 = 2; % Initial concentration of mRNA Y

Kxy = 0.5; % Dissociation constant for X repressing Y transcription

Kyx = 1; % Dissociation constant for Y repressing X transcription

nxy = 5; % Hill coefficient for X repressing Y transcription

nyx = 5; % Hill coefficient for Y repressing X transcription

alpha = 1; % Maximum transcription rate

lambda = 1; % Degradation rate of mRNA transcripts

initial_conditions = [X0 Y0 Kxy Kyx nxy nyx alpha lambda];

% Solve the system of ODEs

[T, y] = ode45(@odefun, t, initial_conditions);

% Plot the results

plot(T, y(:,1:2))

xlabel('Time') % Use lowercase 't' for consistency

ylabel('mRNA Concentration')

legend('X', 'Y')

% System of Differential Equations

function dy = odefun(t, y)

% Extract variables for readability

X = y(1);

Y = y(2);

Kxy = y(3);

Kyx = y(4);

nxy = y(5);

nyx = y(6);

alpha = y(7);

lambda = y(8);

% Equations describing the system (represent mRNA production and degradation)

dX = alpha * 1/(1 + (Y/Kxy)^nxy) - lambda * X;

dY = alpha * (X/Kyx)^nyx / (1 + (X/Kyx)^nyx) - lambda * Y;

% Return vector of derivatives

dy = [dX; dY; 0; 0; 0; 0; 0; 0];

end

**Computer Simulation 14.** Modeling an Oscillatory Gene Network with Two Activators and One Repressor.

The results of this computer simulation are shown in Fig. 3E’.

% Program Description:

% This Matlab program models a three-gene negative feedback loop, wherein gene X activates gene Y,

% gene Y activates gene Z, and gene Z represses gene X. The model is based on differential equations

% that describe the dynamic interactions and regulatory mechanisms among these genes over time.

clear % Clears the workspace

clc % Clears the command window

grid on % Enables grid for the plots

t = 0:.01:20; % Time range from 0 to 20 with a step size of 0.01

% Initial concentrations of genes X, Y, and Z

X0 = 2;

Y0 = 0;

Z0 = 0;

% Model parameters

Kxz = 1; % Repression constant of X by Z

Kyx = 1; % Activation constant of Y by X

Kzy = 1; % Activation constant of Z by Y

nxz = 5; % Hill coefficient for the repression of X by Z

nyx = 5; % Hill coefficient for the activation of Y by X

nzy = 5; % Hill coefficient for the activation of Z by Y

alpha = 2;% Baseline production rate of X, Y, and Z

lambda = 1;% Degradation rate of X, Y, and Z

% Vector of initial conditions and parameters

initial_conditions = [X0 Y0 Z0 Kxz Kyx Kzy nxz nyx nzy alpha lambda];

% Solving the differential equations using ode45

[T, y] = ode45(@odefun, t, initial_conditions);

% Plotting the results

plot(T, [y(:,1:3)])

xlabel 'time'

ylabel 'concentration'

legend('X', 'Y', 'Z')

% grid on

%%%%% Differential Equation %%%%%%

function dy = odefun(t, y)

X = y(1); % Concentration of X

Y = y(2); % Concentration of Y

Z = y(3); % Concentration of Z

Kxz = y(4); % Repression constant of X by Z

Kyx = y(5); % Activation constant of Y by X

Kzy = y(6); % Activation constant of Z by Y

nxz = y(7); % Hill coefficient for the repression of X by Z

nyx = y(8); % Hill coefficient for the activation of Y by X

nzy = y(9); % Hill coefficient for the activation of Z by Y

alpha = y(10);% Baseline production rate of X, Y, and Z

lambda = y(11);% Degradation rate of X, Y, and Z

dX = alpha*1/(1+(Z/Kxz)^nxz) - lambda*X; % Change in X over time

dY = alpha*(X/Kyx)^nyx/(1+(X/Kyx)^nyx) - lambda*Y; % Change in Y over time

dZ = alpha*(Y/Kzy)^nzy/(1+(Y/Kzy)^nzy) - lambda*Z; % Change in Z over time

dy = [dX; dY; dZ; zeros(8,1)]; % Only X, Y, and Z change; other parameters are constant

end

**Computer Simulation 15.** Modeling the Repressilator Network Composed of Three Repressor Genes.

The results of this computer simulation are shown in Fig. 3F’.

% This Matlab program models the repressilator, a genetic regulatory network

% comprising three genes that cyclically repress each other in a negative feedback loop.

% The program uses differential equations to describe the interactions among these genes,

% solving them over time to simulate the oscillatory dynamics. The results are visualized by plotting the concentrations of

% genes X, Y, and Z over time.

clear % Clears the workspace

clc % Clears the command window

grid on % Enables grid for the plots

t = 0:.01:20; % Time range from 0 to 20 with a step size of 0.01

% Initial conditions for gene concentrations

X0 = 2; % Initial concentration of gene X

Y0 = 0; % Initial concentration of gene Y

Z0 = 0; % Initial concentration of gene Z

% Model parameters

Kxz = 1; % Repression constant of X by Z

Kyx = 1; % Repression constant of Y by X

Kzy = 1; % Repression constant of Z by Y

nxz = 5; % Hill coefficient for repression of X by Z

nyx = 5; % Hill coefficient for repression of Y by X

nzy = 5; % Hill coefficient for repression of Z by Y

alpha = 2;% Baseline production rate of the proteins

lambda = 1;% Degradation rate of the proteins

% Vector of initial conditions and parameters

initial_conditions = [X0 Y0 Z0 Kxz Kyx Kzy nxz nyx nzy alpha lambda];

% Solving the differential equations using ode45

[T, y] = ode45(@odefun, t, initial_conditions);

% Plotting the results

plot(T, [y(:,1:3)])

xlabel 'time'

ylabel 'concentration'

legend('X', 'Y', 'Z')

% grid on

%%%%% Differential Equation %%%%%%

function dy = odefun(t, y)

X = y(1); % Concentration of X

Y = y(2); % Concentration of Y

Z = y(3); % Concentration of Z

Kxz = y(4); % Repression constant of X by Z

Kyx = y(5); % Repression constant of Y by X

Kzy = y(6); % Repression constant of Z by Y

nxz = y(7); % Hill coefficient for repression of X by Z

nyx = y(8); % Hill coefficient for repression of Y by X

nzy = y(9); % Hill coefficient for repression of Z by Y

alpha = y(10);% Baseline production rate of the proteins

lambda = y(11);% Degradation rate of the proteins

dX = alpha*1/(1+(Z/Kxz)^nxz) - lambda*X; % Change in X over time

dY = alpha*1/(1+(X/Kyx)^nyx) - lambda*Y; % Change in Y over time

dZ = alpha*1/(1+(Y/Kzy)^nzy) - lambda*Z; % Change in Z over time

dy = [dX; dY; dZ; zeros(8,1)]; % Only X, Y, and Z change; other parameters are constant

end

**Computer Simulation 16.** Modeling a Multi-Stable Gene Regulatory Network with Mutually Repressing Genes.

The results of this computer simulation are shown in Fig. 3G’.

% This Matlab program models a genetic cascade with mutual repressive connections.

% The program uses differential equations to describe the interactions among these genes,

% solving them over time to simulate the dynamics. The results are visualized by plotting

% the concentrations of the genes over time.

clear % Clears the workspace

clc % Clears the command window

grid on % Enables grid for the plots

t = 0:.01:10; % Time range from 0 to 20 with a step size of 0.01

% Initial conditions for gene concentrations

X1_0 = 1.5; % Initial concentration of gene X1

X2_0 = 1; % Initial concentration of gene X2

X3_0 = 0.5; % Initial concentration of gene X3

% Model parameters

theta_s = 0.4; % Given constant theta_s

theta_w = 2.5; % Given constant theta_w

n = 5; % Hill coefficient

lambda = 1; % Degradation rate

% Vector of initial conditions and parameters

initial_conditions = [X1_0 X2_0 X3_0];

% Solving the differential equations using ode45

[T, y] = ode45(@odefun, t, initial_conditions);

% Define the color order

colors = [

49/255, 126/255, 194/255; % Blue

192/255, 56/255, 48/255; % Red

90/255, 170/255, 70/255; % Green

];

% Set the default color order

set(gca, 'ColorOrder', colors, 'NextPlot', 'replacechildren');

% Plotting the results

plot(T, y)

xlabel 'time'

ylabel 'concentration'

legend('X1', 'X2', 'X3')

grid off

%%%%% Differential Equation %%%%%%

function dy = odefun(t, y)

X1 = y(1); % Concentration of X1

X2 = y(2); % Concentration of X2

X3 = y(3); % Concentration of X3

theta_s = 0.4; % Given constant theta_s

theta_w = 2.5; % Given constant theta_w

n = 5; % Hill coefficient

lambda = 1; % Degradation rate

a = 3; % Transcription rate constant

% Differential equations for each gene

dX1 = a * (1 / (1 + (X2/theta_s)^n) * 1 / (1 + (X3/theta_s)^n)) - lambda * X1;

dX2 = a * (1 / (1 + (X1/theta_s)^n) * 1 / (1 + (X3/theta_s)^n)) - lambda * X2;

dX3 = a * (1 / (1 + (X2/theta_s)^n) * 1 / (1 + (X1/theta_s)^n)) - lambda * X3;

dy = [dX1; dX2; dX3];

end

**Computer Simulation 17.** Modeling a Genetic Cascade Illustrating Sequential Gene Activation.

The results of this computer simulation are shown in Fig. 3H’.

% This Matlab program models a genetic cascade with mutual repressive connections.

% The program uses differential equations to describe the interactions among these genes,

% solving them over time to simulate the dynamics. The results are visualized by plotting

% the concentrations of the genes over time.

clear % Clears the workspace

clc % Clears the command window

grid on % Enables grid for the plots

t = 0:.01:10; % Time range from 0 to 20 with a step size of 0.01

% Initial conditions for gene concentrations

X1_0 = 3; % Initial concentration of gene X1

X2_0 = 0; % Initial concentration of gene X2

X3_0 = 0; % Initial concentration of gene X3

% Model parameters

theta_s = 0.4; % Given constant theta_s

theta_w = 2.5; % Given constant theta_w

n = 5; % Hill coefficient

lambda = 1; % Degradation rate

% Vector of initial conditions and parameters

initial_conditions = [X1_0 X2_0 X3_0];

% Solving the differential equations using ode45

[T, y] = ode45(@odefun, t, initial_conditions);

% Define the color order

colors = [

49/255, 126/255, 194/255; % Blue

192/255, 56/255, 48/255; % Red

90/255, 170/255, 70/255; % Green

];

% Set the default color order

set(gca, 'ColorOrder', colors, 'NextPlot', 'replacechildren');

% Plotting the results

plot(T, y)

xlabel 'time'

ylabel 'concentration'

legend('X1', 'X2', 'X3')

%%%%% Differential Equation %%%%%%

function dy = odefun(t, y)

X1 = y(1); % Concentration of X1

X2 = y(2); % Concentration of X2

X3 = y(3); % Concentration of X3

theta_s = 0.4; % Given constant theta_s

theta_w = 2.5; % Given constant theta_w

n = 5; % Hill coefficient

lambda = 1; % Degradation rate

a = 3; % Transcription rate constant

% Differential equations for each gene

dX1 = a * (1 / (1 + (X2/theta_s)^n) * 1 / (1 + (X3/theta_s)^n)) - lambda * X1;

dX2 = a * (1 / (1 + (X1/theta_w)^n) * 1 / (1 + (X3/theta_s)^n)) - lambda * X2;

dX3 = a * (1 / (1 + (X2/theta_w)^n) * 1 / (1 + (X1/theta_s)^n)) - lambda * X3;

dy = [dX1; dX2; dX3];

end

**Computer Simulation 18.** Modeling an Oscillatory Gene Network with Mutual Repression.

The results of this computer simulation are shown in Fig. 3I’.

% This Matlab program models a genetic oscillator with mutual repressive connections.

% The program uses differential equations to describe the interactions among these genes,

% solving them over time to simulate the dynamics. The results are visualized by plotting

% the concentrations of the genes over time.

clear % Clears the workspace

clc % Clears the command window

grid on % Enables grid for the plots

t = 0:.01:10; % Time range from 0 to 20 with a step size of 0.01

% Initial conditions for gene concentrations

X1_0 = 3; % Initial concentration of gene X1

X2_0 = 0; % Initial concentration of gene X2

X3_0 = 0; % Initial concentration of gene X3

% Model parameters

theta_s = 0.4; % Given constant theta_s

theta_w = 2.5; % Given constant theta_w

n = 5; % Hill coefficient

lambda = 1; % Degradation rate

% Vector of initial conditions and parameters

initial_conditions = [X1_0 X2_0 X3_0];

% Solving the differential equations using ode45

[T, y] = ode45(@odefun, t, initial_conditions);

% Define the color order

colors = [

49/255, 126/255, 194/255; % Blue

192/255, 56/255, 48/255; % Red

90/255, 170/255, 70/255; % Green

];

% Set the default color order

set(gca, 'ColorOrder', colors, 'NextPlot', 'replacechildren');

% Plotting the results

plot(T, y)

xlabel 'time'

ylabel 'concentration'

legend('X1', 'X2', 'X3')

%%%%% Differential Equation %%%%%%

function dy = odefun(t, y)

X1 = y(1); % Concentration of X1

X2 = y(2); % Concentration of X2

X3 = y(3); % Concentration of X3

theta_s = 0.4; % Given constant theta_s

theta_w = 2.5; % Given constant theta_w

n = 5; % Hill coefficient

lambda = 1; % Degradation rate

a = 3; % Transcription rate constant

% Differential equations for each gene

dX1 = a * (1 / (1 + (X2/theta_s)^n) ) - lambda * X1;

dX2 = a * (1 / (1 + (X1/theta_w)^n) * 1 / (1 + (X3/theta_s)^n)) - lambda * X2;

dX3 = a * (1 / (1 + (X2/theta_w)^n) * 1 / (1 + (X1/theta_s)^n)) - lambda * X3;

dy = [dX1; dX2; dX3];

end

**Computer Simulation 19.** Gene Regulatory Network Realization of the French Flag Model in a Non-Elongating Tissue.

The results of this computer simulation are shown in Fig. 5C’.

% This MATLAB program simulates a gene regulatory network (GRN) realization of

% the French Flag model in a non-elongating tissue. It models the expression of

% multiple genes in response to a morphogen gradient, demonstrating how different

% activation and repression thresholds can create distinct gene expression patterns

% along a spatial axis. The simulation solves the differential equations governing

% gene regulation over time and space, and plots the resulting gene expression profiles.

clear

clc

close all

%%%%%%%%%%%%%%%%%%%%% PARAMETERS %%%%%%%%%%%%%%%%%%%%%%%%%%%%%%%%%%

a = 0.25; % starting inflection point of the gradient

m = 20; % starting steepness of the gradient

global N; N=4; %number of genes

%%%%%%%%%%%%%%%%%%%%%%%%%%%%%%%%%%%%%%%%%%%%%%%%%%%%%%%%%%%%%%%%%%%%

% Time and spatial axes

t = 0:0.01:5; % time axis

AP_axis = 0:0.001:0.5; % spatial axis

% Initialize solution space

spacetime = zeros(N, length(t), length(AP_axis));

% Solve the model

for pos = 1:length(AP_axis)

initial_conditions = [zeros(1, N) AP_axis(pos) a m];

[~, x] = ode45(@odefun, t, initial_conditions);

spacetime(:, :, pos) = x(:, 1:N)';

end

% Plot the solution

close all

for nt = 1:length(t)

% Setting up the gradient G

u = 0;

current_m = m;

% Gradient G equation

G = 1.5 ./ (1 + exp(-current_m * (AP_axis - a)));

% Plotting

set(gca, 'ColorOrder', ...

[86 128 193; 229 51 50; 79 185 118; 219 152 40; 128 128 128] / 256, ...

'NextPlot', 'replacechildren');

vizScale = .4*[1 1 1 .8]; % to scale expression values for visualization

plot(AP_axis, [vizScale .* squeeze(spacetime(:, nt, :))' G'], 'LineWidth', 5)

axis([0 max(AP_axis) 0 2.5])

axis off

pause(0.0001)

end

%%%%% Model Differential Equations %%%%%%

function dx = odefun(t, x)

global N;

% Passed variables and parameters

x1 = x(1);

x2 = x(2);

x3 = x(3);

x4 = x(4);

AP_axis = x(N+1); % position

a = x(N+2); % starting inflection point of the gradient

m = x(N+3); % starting steepness of the gradient

% Gradient G equation

G = 1.5 / (1 + exp(-m * (AP_axis - a)));

% Gene regulation parameters

w = 2.5; % dissociation constant of weak regulatory interactions

s = 0.2; % dissociation constant of strong regulatory interactions

n = 5; % cooperativity constant

lambda = 1; % decay rate

% Gene regulation equations

dx=zeros(N+3,1);

dx(1) = 3 * (G / .5)^n / (1 + (G / .5)^n) - lambda * x1;

dx(2) = 3 * (G / 1)^n / (1 + (G / 1)^n) - lambda * x2;

dx(3) = 3 * 1 / (1 + (G / .5)^n) - lambda * x3;

dx(4) = 3 * 1 / (1 + (G / 1)^n) - lambda * x4;

end

**Computer Simulation 20.** Defining Gene Expression Boundaries Using Two Morphogen Gradients.

The results of this computer simulation are shown in Fig. 5D’.

% This MATLAB program simulates the definition of gene expression boundaries using

% two morphogen gradients. It models how overlapping gradients can precisely define

% the expression domains of specific genes within a non-elongating tissue. The simulation

% solves the differential equations for gene regulation in response to two gradients

% over time and space, and visualizes the resulting gene expression patterns.

clear

clc

close all

%%%%%%%%%%%%%%%%%%%%% PARAMETERS %%%%%%%%%%%%%%%%%%%%%%%%%%%%%%%%%%

a = 0.25; % starting inflection point of the gradient

m = 10; % starting steepness of the gradient

global N; N=2; %number of genes

%%%%%%%%%%%%%%%%%%%%%%%%%%%%%%%%%%%%%%%%%%%%%%%%%%%%%%%%%%%%%%%%%%%%

% Time and spatial axes

t = 0:0.01:5; % time axis

AP_axis = 0:0.001:0.5; % spatial axis

% Initialize solution space

spacetime = zeros(N, length(t), length(AP_axis));

% Solve the model

for pos = 1:length(AP_axis)

initial_conditions = [0 0 AP_axis(pos) a m];

[~, x] = ode45(@odefun, t, initial_conditions);

spacetime(:, :, pos) = x(:, 1:N)';

end

% Plot the solution

close all

for nt = 1:length(t)

% Gradients G1 and G2 equations

G1 = 1.5 ./ (1 + exp(-m * (AP_axis - a)));

G2 = 1.5 ./ (1 + exp(m * (AP_axis - a)));

% Plotting

set(gca, 'ColorOrder', ...

[86 128 193; 229 51 50; 128 128 128; 1.5*[128 128 128]] / 256, ...

'NextPlot', 'replacechildren');

vizScale = .4*[1 1]; % to scale expression values for visualization

plot(AP_axis, [vizScale .* squeeze(spacetime(:, nt, :))' G1' G2'], 'LineWidth', 5)

axis([0 max(AP_axis) 0 2.5])

axis off

pause(0.0001)

end

%%%%% Model Differential Equations %%%%%%

function dx = odefun(t, x)

global N;

% Passed variables and parameters

x1 = x(1);

x2 = x(2);

AP_axis = x(N+1); % position

a = x(N+2); % starting inflection point of the gradient

m = x(N+3); % starting steepness of the gradient

% Gradients G1 and G2 equations

G1 = 1.5 / (1 + exp(-m * (AP_axis - a)));

G2 = 1.5 / (1 + exp(m * (AP_axis - a)));

% Gene regulation parameters

w = 2.5; % dissociation constant of weak regulatory interactions

s = 0.2; % dissociation constant of strong regulatory interactions

n = 10; % cooperativity constant

lambda = 1; % decay rate

% Gene regulation equations

dx=zeros(N+3,1);

dx(1) = 20 * 1 / (1 + (G1 / .5)^n)* 1 / (1 + (G2 / 1)^n) - lambda * x1;

dx(2) = 20 * 1 / (1 + (G1 / 1)^n)* 1 / (1 + (G2 / .5)^n) - lambda * x2;

end

**Computer Simulation 21.** Gene Regulatory Network Configuration Involving Cross-Regulatory Interactions.

The results of this computer simulation are shown in Fig. 5E’.

% This MATLAB program simulates a gene regulatory network (GRN) configuration involving

% cross-regulatory interactions among genes. It demonstrates how cross-repression between

% genes can refine their expression domains in response to a morphogen gradient, resulting

% in distinct and sharp gene expression patterns across the spatial domain. The simulation

% solves the differential equations governing the GRN over time and space, and plots the

% gene expression profiles.

clear

clc

close all

%%%%%%%%%%%%%%%%%%%%% PARAMETERS %%%%%%%%%%%%%%%%%%%%%%%%%%%%%%%%%%

a = 0.25; % starting inflection point of the gradient

m = 20; % starting steepness of the gradient

%%%%%%%%%%%%%%%%%%%%%%%%%%%%%%%%%%%%%%%%%%%%%%%%%%%%%%%%%%%%%%%%%%%%

% Time and spatial axes

t = 0:0.01:5; % time axis

AP_axis = 0:0.001:0.5; % spatial axis

% Initialize solution space

spacetime = zeros(5, length(t), length(AP_axis));

% Solve the model

for pos = 1:length(AP_axis)

initial_conditions = [.6 zeros(1, 4) AP_axis(pos) a m max(t)];

[~, x] = ode45(@odefun, t, initial_conditions);

spacetime(:, :, pos) = x(:, 1:5)';

end

% Plot the solution

close all

for nt = 1:length(t)

% Setting up the gradient G

u = 0;

current_m = m;

% Gradient G equation

G = 1.5 ./ (1 + exp(-current_m * (AP_axis - a)));

% Plotting

set(gca, 'ColorOrder', ...

[86 128 193; 229 51 50; 79 185 118; 219 152 40; 168 124 78; 128 128 128] / 256, ...

'NextPlot', 'replacechildren');

vizScale = 2*linspace(0.25, 1, 5); % to scale expression values for visualization

plot(AP_axis, [vizScale .* squeeze(spacetime(:, nt, :))' G'], 'LineWidth', 5)

axis([0 max(AP_axis) 0 2.5])

axis off

pause(0.0001)

end

%%%%% Model Differential Equations %%%%%%

function dx = odefun(t, x)

% Passed variables and parameters

x1 = x(1);

x2 = x(2);

x3 = x(3);

x4 = x(4);

x5 = x(5);

AP_axis = x(6); % position

a = x(7); % starting inflection point of the gradient

m = x(8); % starting steepness of the gradient

max_t = x(9); % time of end of simulation

% Setting up the gradient G

u = 0;

current_m = m;

% Gradient G equation

G = 1.5 / (1 + exp(-current_m * (AP_axis - a)));

% Gene regulation parameters

w = 2.5; % dissociation constant of weak regulatory interactions

s = 0.2; % dissociation constant of strong regulatory interactions

sw = 0.4; % dissociation constant of regulatory interactions of intermediate strength

n = 5; % cooperativity constant

lambda = 1; % decay rate

% Gene regulation equations

dx(1) = 3 * (G / 0.2)^n / (1 + (G / 0.2)^n) * 1 / (1 + (x2 / w)^n) * 1 / (1 + (x3 / s)^n) * 1 / (1 + (x4 / s)^n) * 1 / (1 + (x5 / s)^n) - lambda * x1;

dx(2) = 3 * (G / 0.6)^n / (1 + (G / 0.6)^n) * 1 / (1 + (x3 / w)^n) * 1 / (1 + (x4 / s)^n) * 1 / (1 + (x5 / s)^n) - lambda * x2;

dx(3) = 3 * (G / 1)^n / (1 + (G / 1)^n) * 1 / (1 + (x4 / w)^n) * 1 / (1 + (x5 / s)^n) - lambda * x3;

dx(4) = 3 * (G / 1.4)^n / (1 + (G / 1.4)^n) * 1 / (1 + (x5 / sw)^n) - lambda * x4;

dx(5) = 3 * (G / 1.8)^n / (1 + (G / 1.8)^n) - lambda * x5;

% Zero change in parameters

dx(7:9) = 0;

dx = dx';

end

**Computer Simulation 22.** Additive Effect of Separate Enhancers on Gene Expression to Form Periodic Patterns.

The results of this computer simulation are shown in Fig. 5H’.

% This MATLAB program simulates the additive effect of separate enhancers on gene

% expression to form periodic patterns. It models how a gene can be expressed in

% multiple domains, each mediated by different enhancers responding to morphogen gradients.

% The simulation demonstrates how the summation of enhancer activities leads to the overall

% gene expression pattern, and solves the differential equations over time and space to

% visualize the resulting periodic expression.

clear

clc

close all

%%%%%%%%%%%%%%%%%%%%% PARAMETERS %%%%%%%%%%%%%%%%%%%%%%%%%%%%%%%%%%

a = 0.25; % starting inflection point of the gradient

m = 10; % starting steepness of the gradient

global N; N=1; %number of genes

%%%%%%%%%%%%%%%%%%%%%%%%%%%%%%%%%%%%%%%%%%%%%%%%%%%%%%%%%%%%%%%%%%%%

% Time and spatial axes

t = 0:0.01:5; % time axis

AP_axis = 0:0.001:0.5; % spatial axis

% Initialize solution space

spacetime = zeros(N, length(t), length(AP_axis));

% Solve the model

for pos = 1:length(AP_axis)

initial_conditions = [0 AP_axis(pos) a m];

[~, x] = ode45(@odefun, t, initial_conditions);

spacetime(:, :, pos) = x(:, 1:N)';

end

% Plot the solution

close all

for nt = 1:length(t)

% Gradients G1 and G2 equations

G1 = 1.5 ./ (1 + exp(-m * (AP_axis - a)));

G2 = 1.5 ./ (1 + exp(m * (AP_axis - a)));

% Plotting

set(gca, 'ColorOrder', ...

[86 128 193; 128 128 128; 1.5*[128 128 128]] / 256, ...

'NextPlot', 'replacechildren');

vizScale = .4; % to scale expression values for visualization

plot(AP_axis, [vizScale * squeeze(spacetime(:, nt, :)) G1' G2'], 'LineWidth', 5)

axis([0 max(AP_axis) 0 2.5])

axis off

pause(0.0001)

end

%%%%% Model Differential Equations %%%%%%

function dx = odefun(t, x)

global N;

% Passed variables and parameters

x1 = x(1);

AP_axis = x(N+1); % position

a = x(N+2); % starting inflection point of the gradient

m = x(N+3); % starting steepness of the gradient

% Gradients G1 and G2 equations

G1 = 1.5 / (1 + exp(-m * (AP_axis - a)));

G2 = 1.5 / (1 + exp(m * (AP_axis - a)));

% Gene regulation parameters

w = 2.5; % dissociation constant of weak regulatory interactions

s = 0.2; % dissociation constant of strong regulatory interactions

n = 10; % cooperativity constant

lambda = 1; % decay rate

% Gene regulation equations

dx=zeros(N+3,1);

dx(1) = 20 * 1 / (1 + (G1 / .5)^n)* 1 / (1 + (G2 / 1)^n) + 20 * 1 / (1 + (G1 / 1)^n)* 1 / (1 + (G2 / .5)^n) - lambda * x1;

end

**Computer Simulation 23.** Realization of the Speed Regulation Model by Modulating Transcription and Decay Rates.

The results of this computer simulation are shown in Fig. 6B’.

% This MATLAB program realizes the speed regulation model by modulating transcription

% and decay rates. It simulates how a morphogen gradient can regulate the overall transcription

% rate and mRNA degradation rates of multiple genes, thereby controlling the timing of gene

% expression and the progression of cells through different states. The program solves the

% differential equations governing gene expression over time and space, and plots the gene

% expression waves propagating through the tissue.

clc

close all

%%%%%%%%%%%%%%%%%%%%% PARAMETERS %%%%%%%%%%%%%%%%%%%%%%%%%%%%%%%%%%

global a; a = 0.7; % starting inflection point of the gradient

global m; m = 10; % starting steepness of the gradient

global N; N=4; %number of genes

%%%%%%%%%%%%%%%%%%%%%%%%%%%%%%%%%%%%%%%%%%%%%%%%%%%%%%%%%%%%%%%%%%%%

t=0:.01:7; %time axis

AP_axis=0:.001:1; % spatial axis

%solve model

spacetime=zeros(5,length(t),length(AP_axis));

for pos=1:length(AP_axis)

initial_conditions=...

[1*.5+0*.5*exp(-3*AP_axis(pos)) zeros(1,4) AP_axis(pos)];

[~, x]= ode45(@odefun,t,initial_conditions);

spacetime(:,:,pos)=x(:,1:5)';

end

%plot solution

close all

for nt=1:length(t)

% Gradient G equation

G = 1.5 ./ (1 + exp(-m * (AP_axis - a)));

set(gca, 'ColorOrder',...

[86 128 193; 229 51 50; 79 185 118; 219 152 40; 168 124 78;128 128 128 ]/256,...

'NextPlot', 'replacechildren');

plot(AP_axis,[squeeze(spacetime(:,nt,:))' G'],'LineWidth',5)

axis([0 max(AP_axis) 0 2.5])

axis off

pause(.0001)

end

%%%%% Model Differential Equations %%%%%%

function dx = odefun(t,x)

global a

global N;

global m;

%passed variables and parameters

x1=x(1);

x2=x(2);

x3=x(3);

x4=x(4);

x5=x(5);

AP_axis=x(6);%position

% Gradient G equation

G = 1.5 ./ (1 + exp(-m * (AP_axis - a)));

%gene regulation parameters

w=2.5;%dissociation constant of weak regulatory interactions

s=.4;%dissociation constant of strong regulatory interactions

n=5;%cooperativity constant

lambda=1;%decay rate

dx=zeros(6,1);

alpha=1.8;

dx(1)= alpha*G/(1+G)*(1/(1+(x2/s)^n)*1/(1+(x3/s)^n)*1/(1+(x4/s)^n)*1/(1+(x5/s)^n)-lambda*x1);

dx(2)= alpha*G/(1+G)*(1/(1+(x1/w)^n)*1/(1+(x3/s)^n)*1/(1+(x4/s)^n)*1/(1+(x5/s)^n)-lambda*x2);

dx(3)= alpha*G/(1+G)*(1/(1+(x1/s)^n)*1/(1+(x2/w)^n)*1/(1+(x4/s)^n)*1/(1+(x5/s)^n)-lambda*x3);

dx(4)= alpha*G/(1+G)*(1/(1+(x1/s)^n)*1/(1+(x2/s)^n)*1/(1+(x3/w)^n)*1/(1+(x5/s)^n)-lambda*x4);

dx(5)= alpha*G/(1+G)*(1/(1+(x1/s)^n)*1/(1+(x2/s)^n)*1/(1+(x3/s)^n)*1/(1+(x4/w)^n)-lambda*x5);

end

**Computer Simulation 24.** Enhancer Switching Model as a Gene Regulatory Network Realization of the Speed Regulation Model.

The results of this computer simulation are shown in Fig. 6C’.

% This MATLAB program implements the enhancer switching model as a gene regulatory network

% (GRN) realization of the speed regulation model. It simulates how each gene is regulated

% by both a dynamic enhancer (driving sequential activities) and a static enhancer (stabilizing

% expression). The morphogen gradient modulates the balance between the dynamic and static

% enhancers, thereby controlling the speed of gene expression changes. The program solves

% the differential equations over time and space, and visualizes the resulting gene expression

% patterns.

clc

close all

%%%%%%%%%%%%%%%%%%%%% PARAMETERS %%%%%%%%%%%%%%%%%%%%%%%%%%%%%%%%%%

global a; a = 0.7; % starting inflection point of the gradient

global m; m = 5*1; % starting steepness of the gradient

global N; N=4; %number of genes

%%%%%%%%%%%%%%%%%%%%%%%%%%%%%%%%%%%%%%%%%%%%%%%%%%%%%%%%%%%%%%%%%%%%

t=0:.01:10; %time axis

AP_axis=0:.001:1; % spatial axis

%solve model

spacetime=zeros(5,length(t),length(AP_axis));

for pos=1:length(AP_axis)

initial_conditions=...

[.5+0*.1*exp(-3*AP_axis(pos)) zeros(1,4) AP_axis(pos)];

[~, x]= ode45(@odefun,t,initial_conditions);

spacetime(:,:,pos)=x(:,1:5)';

end

%plot solution

close all

for nt=1:length(t)

% Gradient G equation

G = 1.5 ./ (1 + exp(-m * (AP_axis - a)));

set(gca, 'ColorOrder',...

[86 128 193; 229 51 50; 79 185 118; 219 152 40; 168 124 78;128 128 128 ]/256,...

'NextPlot', 'replacechildren');

plot(AP_axis,[squeeze(spacetime(:,nt,:))' G'],'LineWidth',5)

axis([0 max(AP_axis) 0 2.5])

axis off

pause(.0001)

end

%%%%% Model Differential Equations %%%%%%

function dx = odefun(t,x)

global a

global N;

global m;

%passed variables and parameters

x1=x(1);

x2=x(2);

x3=x(3);

x4=x(4);

x5=x(5);

AP_axis=x(6);%position

% Gradient G equation

G = 1.5 ./ (1 + exp(-m * (AP_axis - a)));

%gene regulation parameters

w=2.5;%dissociation constant of weak regulatory interactions

s=.4;%dissociation constant of strong regulatory interactions

n=5;%cooperativity constant

lambda=1;%decay rate

nn=1;

%dynamic module

dynamic(1)= G^nn/(1+G^nn)*1/(1+(x2/s)^n)*1/(1+(x3/s)^n)*1/(1+(x4/s)^n)*1/(1+(x5/s)^n);

dynamic(2)= G^nn/(1+G^nn)*1/(1+(x1/w)^n)*1/(1+(x3/s)^n)*1/(1+(x4/s)^n)*1/(1+(x5/s)^n);

dynamic(3)= G^nn/(1+G^nn)*1/(1+(x1/s)^n)*1/(1+(x2/w)^n)*1/(1+(x4/s)^n)*1/(1+(x5/s)^n);

dynamic(4)= G^nn/(1+G^nn)*1/(1+(x1/s)^n)*1/(1+(x2/s)^n)*1/(1+(x3/w)^n)*1/(1+(x5/s)^n);

dynamic(5)= G^nn/(1+G^nn)*1/(1+(x1/s)^n)*1/(1+(x2/s)^n)*1/(1+(x3/s)^n)*1/(1+(x4/w)^n);

%static module

static(1)= 1/(1+G^nn)*1/(1+(x2/s)^n)*1/(1+(x3/s)^n)*1/(1+(x4/s)^n)*1/(1+(x5/s)^n);

static(2)= 1/(1+G^nn)*1/(1+(x1/s)^n)*1/(1+(x3/s)^n)*1/(1+(x4/s)^n)*1/(1+(x5/s)^n);

static(3)= 1/(1+G^nn)*1/(1+(x1/s)^n)*1/(1+(x2/s)^n)*1/(1+(x4/s)^n)*1/(1+(x5/s)^n);

static(4)= 1/(1+G^nn)*1/(1+(x1/s)^n)*1/(1+(x2/s)^n)*1/(1+(x3/s)^n)*1/(1+(x5/s)^n);

static(5)= 1/(1+G^nn)*1/(1+(x1/s)^n)*1/(1+(x2/s)^n)*1/(1+(x3/s)^n)*1/(1+(x4/s)^n);

%total gene regulation

dx=zeros(6,1);

c=2;

d=3;

dx(1)= c*static(1)+d*dynamic(1)-lambda*x1;

dx(2)= c*static(2)+d*dynamic(2)-lambda*x2;

dx(3)= c*static(3)+d*dynamic(3)-lambda*x3;

dx(4)= c*static(4)+d*dynamic(4)-lambda*x4;

dx(5)= c*static(5)+d*dynamic(5)-lambda*x5;

end

**Computer Simulation 25.** Impact of an Overactive Enhancer in the Additive Integration Model.

The results of this computer simulation are shown in Fig. 7A’ (WT; parameter mut_flag=0 in the program) and 7A’’ (overactive dynamic enhancer of Gene 3; parameter mut_flag=1 in the program).

% This MATLAB program investigates the impact of an overactive enhancer in the additive

% integration model. It simulates the enhancer switching model where the transcriptional

% contributions of enhancers are additive. The program demonstrates how an overactive

% dynamic enhancer can disrupt the overall function of the gene regulatory network (GRN),

% leading to aberrant gene expression patterns. It solves the differential equations over

% time and space, and plots the gene expression profiles under both wild-type and mutant

% conditions.

clc

close all

%%%%%%%%%%%%%%%%%%%%% PARAMETERS %%%%%%%%%%%%%%%%%%%%%%%%%%%%%%%%%%

global a; a = 0.7; % starting inflection point of the gradient

global m; m = 5*1; % starting steepness of the gradient

global N; N=5; %number of genes

global mut_flag; % 0 for WT, 1 for overactive dynamic enhancer of Gene 3

mut_flag=1;

%%%%%%%%%%%%%%%%%%%%%%%%%%%%%%%%%%%%%%%%%%%%%%%%%%%%%%%%%%%%%%%%%%%%

t=0:.01:9; %time axis

AP_axis=0:.001:1*1; % spatial axis

%solve model

spacetime=zeros(5,length(t),length(AP_axis));

for pos=1:length(AP_axis)

initial_conditions=...

[.5+0*.1*exp(-3*AP_axis(pos)) zeros(1,4) AP_axis(pos)];

[~, x]= ode45(@odefun,t,initial_conditions);

spacetime(:,:,pos)=x(:,1:5)';

end

max_spacetime=max(max(max(spacetime)));

%plot solution

close all

for nt=1:length(t)

% Gradient G equation

G = 1.5 ./ (1 + exp(-m * (AP_axis - a)));

set(gca, 'ColorOrder',...

[86 128 193; 229 51 50; 79 185 118; 219 152 40; 168 124 78;128 128 128 ]/256,...

'NextPlot', 'replacechildren');

plot(AP_axis,[squeeze(spacetime(:,nt,:))' G'],'LineWidth',5)

axis([0 max(AP_axis) 0 max([max_spacetime 1.5])])

axis off

pause(.0001)

end

%%%%% Model Differential Equations %%%%%%

function dx = odefun(t,x)

global a

global N;

global m;

global mut_flag;

%passed variables and parameters

x1=x(1);

x2=x(2);

x3=x(3);

x4=x(4);

x5=x(5);

AP_axis=x(6);%position

% Gradient G equation

G = 1.5 ./ (1 + exp(-m * (AP_axis - a)));

%gene regulation parameters

w=2.5;%dissociation constant of weak regulatory interactions

s=.4;%dissociation constant of strong regulatory interactions

n=5;%cooperativity constant

lambda=1;%decay rate

nn=1;

%dynamic module

dynamic(1)= G^nn/(1+G^nn)*1/(1+(x2/s)^n)*1/(1+(x3/s)^n)*1/(1+(x4/s)^n)*1/(1+(x5/s)^n);

dynamic(2)= G^nn/(1+G^nn)*1/(1+(x1/w)^n)*1/(1+(x3/s)^n)*1/(1+(x4/s)^n)*1/(1+(x5/s)^n);

dynamic(3)= G^nn/(1+G^nn)*1/(1+(x1/s)^n)*1/(1+(x2/w)^n)*1/(1+(x4/s)^n)*1/(1+(x5/s)^n);

dynamic(4)= G^nn/(1+G^nn)*1/(1+(x1/s)^n)*1/(1+(x2/s)^n)*1/(1+(x3/w)^n)*1/(1+(x5/s)^n);

dynamic(5)= G^nn/(1+G^nn)*1/(1+(x1/s)^n)*1/(1+(x2/s)^n)*1/(1+(x3/s)^n)*1/(1+(x4/w)^n);

%static module

static(1)= 1/(1+G^nn)*1/(1+(x2/s)^n)*1/(1+(x3/s)^n)*1/(1+(x4/s)^n)*1/(1+(x5/s)^n);

static(2)= 1/(1+G^nn)*1/(1+(x1/s)^n)*1/(1+(x3/s)^n)*1/(1+(x4/s)^n)*1/(1+(x5/s)^n);

static(3)= 1/(1+G^nn)*1/(1+(x1/s)^n)*1/(1+(x2/s)^n)*1/(1+(x4/s)^n)*1/(1+(x5/s)^n);

static(4)= 1/(1+G^nn)*1/(1+(x1/s)^n)*1/(1+(x2/s)^n)*1/(1+(x3/s)^n)*1/(1+(x5/s)^n);

static(5)= 1/(1+G^nn)*1/(1+(x1/s)^n)*1/(1+(x2/s)^n)*1/(1+(x3/s)^n)*1/(1+(x4/s)^n);

%total gene regulation

dx=zeros(6,1);

mut=1;

if(mut_flag)

mut=3;

end

C=1*[2 2 2*1 2 2];

D=1*[3 3 3*mut 3 3];

%%% Additive

dx(1)= C(1)*static(1)+D(1)*dynamic(1)-lambda*x1;

dx(2)= C(2)*static(2)+D(2)*dynamic(2)-lambda*x2;

dx(3)= C(3)*static(3)+D(3)*dynamic(3)-lambda*x3;

dx(4)= C(4)*static(4)+D(4)*dynamic(4)-lambda*x4;

dx(5)= C(5)*static(5)+D(5)*dynamic(5)-lambda*x5;

end

**Computer Simulation 26.** Mitigation of Overactive Enhancer Effects Using Enhancer Competition Integration.

The results of this computer simulation are shown in Fig. 7B’ (WT; parameter mut_flag=0 in the program) and 7B’’ (overactive dynamic enhancer of Gene 3; parameter mut_flag=1 in the program).

% This MATLAB program demonstrates the mitigation of overactive enhancer effects using

% enhancer competition integration. It simulates the enhancer switching model where enhancers

% compete for binding to the promoter, normalizing the transcriptional output. The program

% shows how the competitive integration mechanism preserves the overall performance of the

% gene regulatory network (GRN) even in the presence of an overactive enhancer. It solves

% the differential equations over time and space, and visualizes the gene expression patterns

% under both wild-type and mutant conditions.

clc

close all

%%%%%%%%%%%%%%%%%%%%% PARAMETERS %%%%%%%%%%%%%%%%%%%%%%%%%%%%%%%%%%

global a; a = 0.7; % starting inflection point of the gradient

global m; m = 5*1; % starting steepness of the gradient

global N; N=5; %number of genes

global mut_flag; % 0 for WT, 1 for overactive dynamic enhancer of Gene 3

mut_flag=0;

%%%%%%%%%%%%%%%%%%%%%%%%%%%%%%%%%%%%%%%%%%%%%%%%%%%%%%%%%%%%%%%%%%%%

t=0:.01:9; %time axis

AP_axis=0:.001:1*1; % spatial axis

%solve model

spacetime=zeros(5,length(t),length(AP_axis));

for pos=1:length(AP_axis)

initial_conditions=...

[.5+0*.1*exp(-3*AP_axis(pos)) zeros(1,4) AP_axis(pos)];

[~, x]= ode45(@odefun,t,initial_conditions);

spacetime(:,:,pos)=x(:,1:5)';

end

max_spacetime=max(max(max(spacetime)));

%plot solution

close all

for nt=1:length(t)

% Gradient G equation

G = 1.5 ./ (1 + exp(-m * (AP_axis - a)));

set(gca, 'ColorOrder',...

[86 128 193; 229 51 50; 79 185 118; 219 152 40; 168 124 78;128 128 128 ]/256,...

'NextPlot', 'replacechildren');

plot(AP_axis,[squeeze(spacetime(:,nt,:))' G'],'LineWidth',5)

axis([0 max(AP_axis) 0 max([max_spacetime 1.5])])

axis off

pause(.0001)

end

%%%%% Model Differential Equations %%%%%%

function dx = odefun(t,x)

global a

global N;

global m;

global mut_flag;

%passed variables and parameters

x1=x(1);

x2=x(2);

x3=x(3);

x4=x(4);

x5=x(5);

AP_axis=x(6);%position

% Gradient G equation

G = 1.5 ./ (1 + exp(-m * (AP_axis - a)));

%gene regulation parameters

w=2.5;%dissociation constant of weak regulatory interactions

s=.4;%dissociation constant of strong regulatory interactions

n=5;%cooperativity constant

lambda=1;%decay rate

nn=1;

%dynamic module

dynamic(1)= G^nn/(1+G^nn)*1/(1+(x2/s)^n)*1/(1+(x3/s)^n)*1/(1+(x4/s)^n)*1/(1+(x5/s)^n);

dynamic(2)= G^nn/(1+G^nn)*1/(1+(x1/w)^n)*1/(1+(x3/s)^n)*1/(1+(x4/s)^n)*1/(1+(x5/s)^n);

dynamic(3)= G^nn/(1+G^nn)*1/(1+(x1/s)^n)*1/(1+(x2/w)^n)*1/(1+(x4/s)^n)*1/(1+(x5/s)^n);

dynamic(4)= G^nn/(1+G^nn)*1/(1+(x1/s)^n)*1/(1+(x2/s)^n)*1/(1+(x3/w)^n)*1/(1+(x5/s)^n);

dynamic(5)= G^nn/(1+G^nn)*1/(1+(x1/s)^n)*1/(1+(x2/s)^n)*1/(1+(x3/s)^n)*1/(1+(x4/w)^n);

%static module

static(1)= 1/(1+G^nn)*1/(1+(x2/s)^n)*1/(1+(x3/s)^n)*1/(1+(x4/s)^n)*1/(1+(x5/s)^n);

static(2)= 1/(1+G^nn)*1/(1+(x1/s)^n)*1/(1+(x3/s)^n)*1/(1+(x4/s)^n)*1/(1+(x5/s)^n);

static(3)= 1/(1+G^nn)*1/(1+(x1/s)^n)*1/(1+(x2/s)^n)*1/(1+(x4/s)^n)*1/(1+(x5/s)^n);

static(4)= 1/(1+G^nn)*1/(1+(x1/s)^n)*1/(1+(x2/s)^n)*1/(1+(x3/s)^n)*1/(1+(x5/s)^n);

static(5)= 1/(1+G^nn)*1/(1+(x1/s)^n)*1/(1+(x2/s)^n)*1/(1+(x3/s)^n)*1/(1+(x4/s)^n);

%total gene regulation

dx=zeros(6,1);

mut=1;

if(mut_flag)

mut=3;

end

C=1*[2 2 2*1 2 2];

D=1*[3 3 3*mut 3 3];

%%% Competitive: ke1 and ke2 -> kt

ne=1;

dx(1)= (C(1)*static(1)+D(1)*dynamic(1))^ne/(1+(C(1)*static(1)+D(1)*dynamic(1))^ne)-lambda*x1;

dx(2)= (C(2)*static(2)+D(2)*dynamic(2))^ne/(1+(C(2)*static(2)+D(2)*dynamic(2))^ne)-lambda*x2;

dx(3)= (C(3)*static(3)+D(3)*dynamic(3))^ne/(1+(C(3)*static(3)+D(3)*dynamic(3))^ne)-lambda*x3;

dx(4)= (C(4)*static(4)+D(4)*dynamic(4))^ne/(1+(C(4)*static(4)+D(4)*dynamic(4))^ne)-lambda*x4;

dx(5)= (C(5)*static(5)+D(5)*dynamic(5))^ne/(1+(C(5)*static(5)+D(5)*dynamic(5))^ne)-lambda*x5;

end
