# Supplementary figures and images for: From genes to patterns: a framework for modeling the emergence of embryonic development from transcriptional regulation
Source: Front Cell Dev Biol. 2025 Mar 20;13:1522725. doi: 10.3389/fcell.2025.1522725 (PMC11966961; doi:10.3389/fcell.2025.1522725)

Figure S1

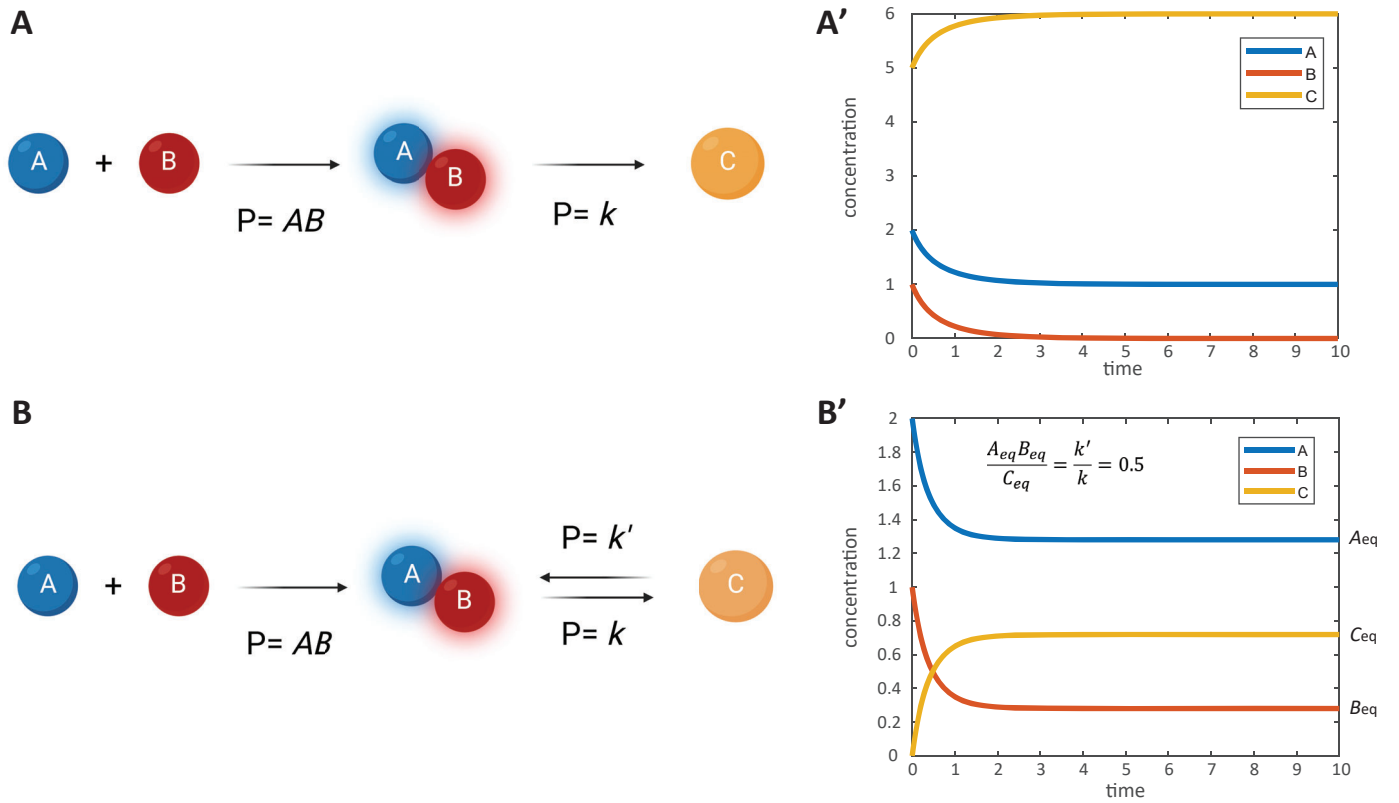

Supplement: Supplementary file 2 [file Supplementaryfile1.pdf]

Figure S2

A

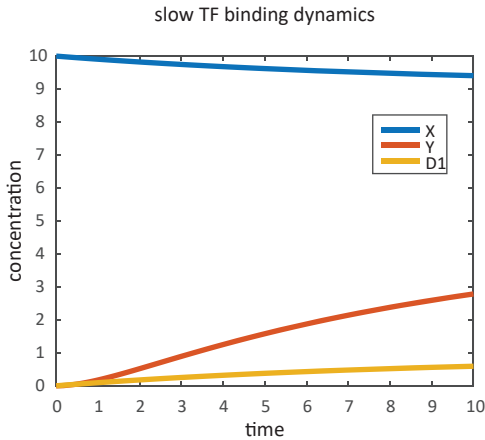

B

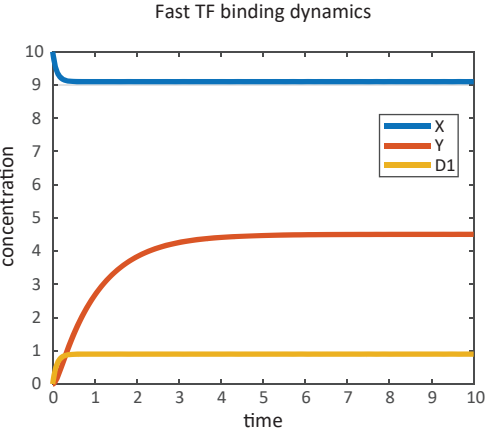

C

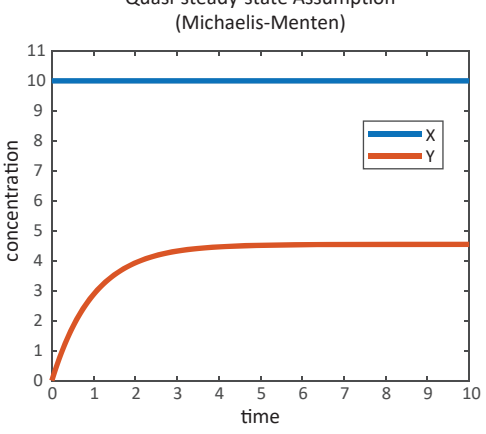

Supplement: Supplementary file 3 [file Supplementaryfile2.pdf]

Figure S3

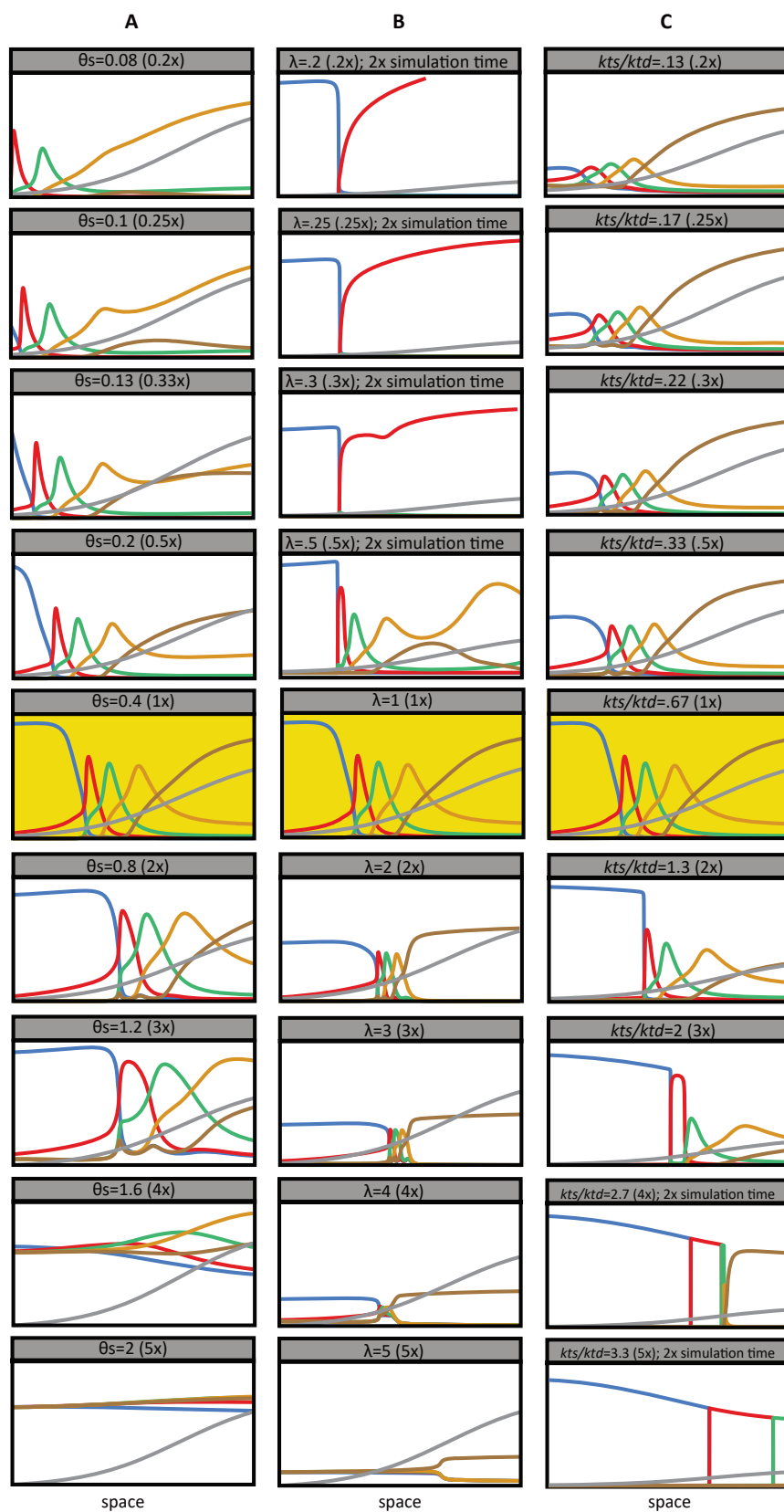

Supplement: Supplementary file 5 [file Supplementaryfile3.pdf]
