# Supplementary material for: From genes to patterns: a framework for modeling the emergence of embryonic development from transcriptional regulation
Source: Front Cell Dev Biol. 2025 Mar 20;13:1522725. doi: 10.3389/fcell.2025.1522725 (PMC11966961; doi:10.3389/fcell.2025.1522725)
Supplement: Supplementary file 4 [file Supplementaryfile4.docx]

**Text S1**

**Modeling chemical reactions**

The Law of Mass Action describes the relationship between the concentrations of reactants and the reaction rate in a chemical reaction. According to this law, the reaction rate is directly proportional to the product of the reactants' concentrations. In this context, the concentration of a species can also be understood as the probability of a molecule encountering another molecule with which it can react.

In the example shown in Fig. S1-A, chemical species A interacts with chemical species B to produce a new species C, with a rate constant *k*. This constant can be interpreted as the probability that, upon collision, species A and B will successfully transform into species C. This relationship is represented by the following chemical equation.

$$\begin{aligned} A+B\underset{\to}{k}C \#S1-1 \end{aligned}$$

According to the law of mass action, the rate at which this forward reaction occurs can be described by *kAB*, where *k* is the rate constant, and A and B represent the concentrations of the reactants. The rates of change of the concentrations of the reactants and the product can be described by the following system of differential equations:

$$\begin{aligned} \frac{dA}{dt}=\frac{dB}{dt}=-\frac{dC}{dt}=-kAB \#S1-2 \end{aligned}$$

As the reaction proceeds, the concentrations of A and B decrease as they are consumed to form the product C, as shown in Computer Simulation 1 (Text S2) and Fig. S1-A’.

*Equilibrium*

Here we will consider a reversible version of the reaction considered in the example above:

$$\begin{aligned} A+B {\underset{\to}{k} \atop\overset{\leftarrow}{k^{'}}} C \#S1-3 \end{aligned}$$

$$\begin{aligned} \frac{dA}{dt}=\frac{dB}{dt}=-\frac{dC}{dt}=-kAB+k^{'}C \#S1-4 \end{aligned}$$

In a reversible reaction, the system will eventually reach equilibrium, where the rates of the forward and reverse reactions are equal. At equilibrium, the concentrations of the reactants and product remain constant because the forward reaction rate equals the reverse reaction rate:

$$\begin{aligned} \frac{dA}{dt}=\frac{dB}{dt}=\frac{dC}{dt}=0 \#S1-5 \end{aligned}$$

Substituting this into Eq. S1-2, the relative relationship between reactants at equilibrium can be given by Eq. S1-6, as shown in by Computer Simulation 2 (Text S2) and Fig. S1-B’.

$$\begin{aligned} \frac{A_{eq}B_{eq}}{C_{eq}}=\frac{k^{'}}{k} \#S1-6 \end{aligned}$$
